# Differences in Gut Microbial and Serum Biochemical Indices Between Sows With Different Productive Capacities During Perinatal Period

**DOI:** 10.3389/fmicb.2019.03047

**Published:** 2020-01-17

**Authors:** Yirui Shao, Jian Zhou, Xia Xiong, Lijun Zou, Xiangfeng Kong, Bie Tan, Yulong Yin

**Affiliations:** ^1^Key Laboratory of Agro-Ecological Processes in Subtropical Region, National Engineering Laboratory for Pollution Control and Waste Utilization in Livestock and Poultry Production, Hunan Provincial Engineering Research Center for Healthy Livestock and Poultry Production, Scientific Observing and Experimental Station of Animal Nutrition and Feed Science in South-Central, Ministry of Agriculture, Institute of Subtropical Agriculture, Chinese Academy of Sciences, Changsha, China; ^2^University of Chinese Academy of Sciences, Beijing, China; ^3^Laboratory of Basic Biology, Hunan First Normal University, Changsha, China; ^4^Hunan International Joint Laboratory of Animal Intestinal Ecology and Health, Laboratory of Animal Nutrition and Human Health, College of Life Sciences, Hunan Normal University, Changsha, China

**Keywords:** gut microbiota, productive capacity, perinatal period, sows, serum immunity

## Abstract

Maternal gut microflora changes dramatically during perinatal period and plays a vital role in animal health and reproductive performance. However, little is known about the microbial differences between sows with different productive capacities during perinatal period. Hence, this study explored fecal microbial diversity, composition, metabolic functions, and phenotypes differences between high productive capacity (HPC, litter size ≥ 15) and low productive capacity (LPC, litter size ≤ 7) sows during late pregnancy (LP, the third day before due date) and early stage after parturition (EAP, the third day after parturition) as well as serum biochemical indices differences after parturition. Results showed that HPC sows had lower microbial richness at LP stage and higher microbial diversity at EAP stage than LPC sows. Several genera belonging to the *Prevotellaceae* family exhibited higher abundance, while some genera belonging to the *Ruminococcaceae* family exhibited lower abundance in HPC sows compared to LPC sows at LP stage. Moreover, the relative abundance of *Eubacterium_coprostanoligenes_group* and *Ruminococcaceae_UCG-014* in HPC sows was significantly higher than that in LPC sows at EAP stage. The predicted metabolic functions related to Lipopolysaccharide biosynthesis were significantly higher in HPC sows at LP stage. Further, HPC sows had significantly higher blood urea nitrogen (BUN) and high-density lipoprotein cholesterol (HDL-C) levels after parturition, and there were strong correlations between BUN level and the relative abundance of genera belonging to the *Ruminococcaceae* families. These results indicated that the HPC sows may experience greater inflammation than LPC sows at LP stage. Inflammation environment might impact health but promote parturition. The microbial differences at EAP stage might be beneficial to hemostasis and anti-inflammation, which might contribute to postpartum recovery in HPC sow.

## Introduction

Pregnant mothers undergo various changes in immunity, metabolism, steroid hormone production and behaviors (Linzer and Fisher, 1999). Uterine contraction, pain and the increasing level of plasma cortisol contribute to physiological stresses, which might lead to dysregulated secretion of oxytocin, oxidative stress, liver damage and impaired immunity (Lawrence et al., 1992; van de Ligt et al., 2002; Verheyen et al., 2007; Jarvis et al., 2010; Marek et al., 2013). Further, some females develop the metabolic syndrome such as decreased insulin sensitivity in late pregnancy (Barbour et al., 2007). Reduced insulin sensitivity may lead to decreased feed intake in sows during lactation (Père and Etienne, 2007). The perinatal period (late pregnancy, LP and early stage after parturition, EAP) is a critical stage for sows, during which most of piglets died under unreasonable management (Bäckström, 1973). However, supervision and nutritional feed supplements can improve sow health and reproductive performance (Holyoake et al., 1995; Kim et al., 2007). Thus, changes that occur in sows during perinatal period need to be further explored, which will guide human interventions to improve sow reproductivity.

The microbiome is of significant importance to a host, which affects the host’s immune system, metabolism, emotion and cognition (Chu and Mazmanian, 2013; Rothschild et al., 2018; Sarkar et al., 2018). Gestation changes the microbiota structure, while intestinal microbes remain stable during lactation (Liu et al., 2019). It was reported that intestinal microflora changed dramatically throughout pregnancy (Koren et al., 2012). In particular, stools from the third trimester contained lower microbial diversity and a higher abundance of *Proteobacteria* and *Actinobacteria*, which contributed to increased adiposity and insulin insensitivity (Koren et al., 2012). Regarding the host’s immune system, SCFAs produced by microbiota are critical for epithelial barrier function, tumor suppression, antioxidation, cytokine production and anti-inflammatory effects (Maslowski and Mackay, 2011; Maynard et al., 2012). Moreover, microflora plays a vital role in reproductive performance. Probiotics feed supplements have been shown to improve sow reproductive capacity by influencing gut microbiota (Alexopoulos et al., 2004; Tsukahara et al., 2018; Cao et al., 2019).

The gut microbiota is a center regulator of host metabolism (Schoeler and Caesar, 2019). The gut-liver axis enables the host to control and shape the gut microbiota and affect animal’s feeding behavior and energy metabolism (Ringseis et al., 2019). Gut microbiota undergo a significant shift during pregnancy (Santacruz et al., 2010; Huang et al., 2019). Santacruz et al. (2010) showed that gut microbiota is associated with body weight, weight gain and biochemical parameters in pregnant women. Huang et al. (2019) showed that host-microbiota interactions during the perinatal period impact host metabolism of sows. However, gut microbial differences between sows with different productive capacities and its relationship with serum biochemical indices remain elusive.

Therefore, this study investigated differences in gut microbiota between low productive capacity (LPC, litter size ≤ 7) and high productive capacity (HPC, litter size ≥ 15) sows during perinatal period. Further, health status between sows with different reproductivity after parturition was compared using serum biochemical indices to determine its correlations with microbiota.

## Materials and Methods

### Experimental Design and Ethics Statement

This experiment followed guidelines for animal research approved by the Animal Welfare Committee of the Institute of Subtropical Agriculture, Chinese Academy of Sciences. The feeding experiments were performed at the pig breeding farm in Hunan province, which had no history of serious bacterial or viral infections. Eighty multiparous hybrid pregnant sows (Landrace × Yorkshire) were used in the experiments. They had similar expected dates of confinement, backfat thicknesses and no medical history. They were in 3–6 birth order and the same physical condition. After parturition, 6 HPC sows (litter size ≥ 15) and 6 LPC sows (litter size ≤ 7) were chosen according to their litter sizes (Supplementary Table S1) and they all received the amoxicillin treatment to diminish postpartum inflammation for 3 days. All sows were provided with the same commercial formula feed once at LP stage and twice after parturition every day. Sows were raised individually in a piggery with hard plastic slatted flooring and they had free access to water through nipple drinkers.

### Sample Collection

At the third day before due date (late stage of pregnancy), feces of all the 80 sows were collected. And feces of 6 HPC as well as 6 LPC sows were collected at the third day after parturition (early stage after parturition). Sterile centrifugal tubes were used to collect fresh feces, which were immediately frozen in liquid nitrogen, and stored at −80°C. Samples were grouped as follows: A, C: sows with high productive capacity (HPC) at LP and EAP stage separately; B, D: sows with low productive capacity (LPC) at LP and EAP stage separately. About 5mL of blood was collected the day after parturition via the auricular vein with vacuum tubes and centrifuged at 1500 *g* for 15 min. Subsequently, the supernatant was stored at −20°C before further determination.

### Microbiota Analysis Based on 16S RNA High-Throughput Sequencing

Microbiota analysis was conducted using 6 fecal samples in each group from different sows and about 0.25 g of each was used to extract bacterial DNA using CTAB/SDA method. The composition and diversity of microflora were analyzed by 16S rRNA high-throughput sequencing. 16S rRNA genes of V4 regions were amplified using 515F: 5′-GTGCCAGCMGCCGCGGTAA-3′ and 806R: 5′-GGACTACHVGGGTWTCTAAT-3′ primers with barcodes. Illumina HiSeq 2500 platform was used to conduct paired-end sequencing. Raw tags were assembled and filtered under specific conditions (Bokulich et al., 2013) to obtain clean data using the QIIME (V1.7.0) (Caporaso et al., 2010) and FLASH (V1.2.7) (Tanja and Salzberg, 2011). Sequences were analyzed and operational taxonomic units (OUT) were determined using UPARSE (v7.0.1001) (Edgar, 2013). Sequences were assigned to the same OTUs at a 97% similarity level. The GreenGene Database^1^ (Desantis et al., 2006) based on RDP classifier (V 2.2)^2^ (Qiong et al., 2007) was used to assigned sequences to a taxonomic level. The assembled HiSeq sequences obtained in the present study were submitted to NCBI Sequence Read Archive (SRA, No. PRJNA565644).

### Metagenome Prediction, Functional Metabolic Pathways and Metabolic Phenotypes Analysis

Functional metagenomes were predicted using PICRUSt (V1.1.3) (Langille et al., 2013). OTUs were verified using the Genome Prediction Tutorial for PICRUSt. Normalized 16S rRNA data were analyzed to predict metagenomes using the database of Kyoto Encyclopedia of Genes and Genomes (KEGG) Orthology^3^. Functional differences were explored using STAMP (V2.1.3) (Parks et al., 2014). Additionally, microbial phenotypes were predicted using Bugbase^4^ based on 16S RNA data and mapping files following provided instructions (Ward et al., 2017b).

### Determination of Serum Biochemical Indices and Their Correlations With Microbial Abundance

Serum biochemical parameters including blood urea nitrogen (BUN), creatinine (CREA), triglyceride (TG), high-density lipoprotein cholesterol (HDL-C), low-density lipoprotein cholesterol (LDL-C), glucose (GLU), AST, ALT, total protein (TP), albumin (ALB), globulin (GLO), ration of albumin/globulin (A/G), and cholesterol (CHO) were determined by TBA-120FR biochemistry analyzer (Toshiba Medical Systems Corporation, Tokyo, Japan). IgM, IgA and IgG were analyzed using an enzyme-linked immunosorbent assay kit (Cusabio Biotech Co., Hubei, China) following provided instructions. The correlations between microbial abundance at the genus level and serum biochemical indices were evaluated by Spearman’s correlation analysis and visualized diagrams were created using R (V2.15.3).

### Statistical Analysis

Statistical analyses were performed using SPASS 22 (SPSS Inc.). Alpha and beta community diversity were calculated with QIIME (V1.7.0). R (V2.15.3) and GraphPad Prism (V8.0.2) were used to create visualized diagrams. For PICRUSt results, differences between groups were analyzed by one-way ANOVA and the Tukey–Kramer multiple comparisons test. Paired *T*-test (LP vs. EAP) or independent *T*-test (HPC vs. LPC) was used after accessing normality with Shapiro–Wilk *W*-test to analyze microbial alpha diversity and serum biochemical indices. Variability in the data was expressed as means ± SD, and level of *P* < 0.05 was considered significant. Wilcoxon signed-rank test (LP vs. EAP) or Mann–Whitney *U*-test (HPC vs. LPC) was applied to analyze gut microbial phenotypes differences.

## Results

### Diversity Changes in Gut Microbiota

**FIGURE 1:**
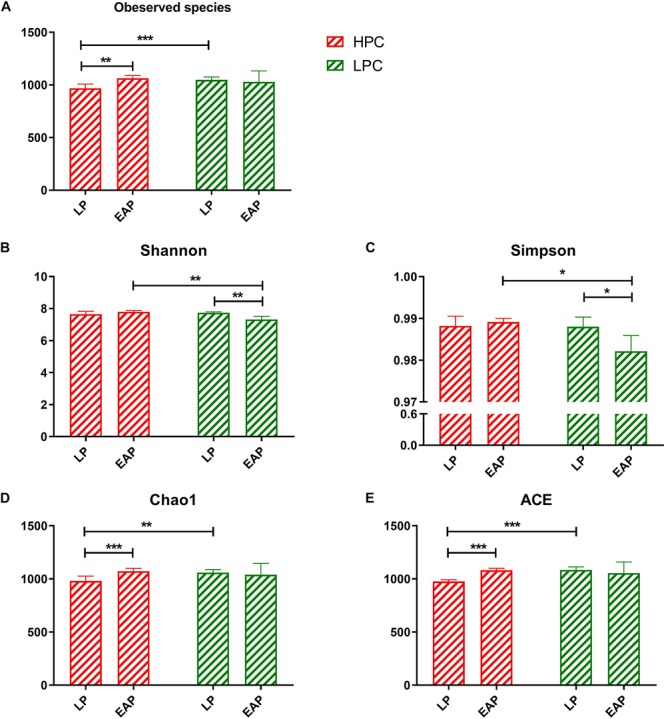
Alpha diversity and richness of gut microbiota from high productive capacity (HPC) and low productive capacity (LPC) sows at late pregnancy (LP) and early stage after parturition (EAP) stage. **(A–E)** Indices for alpha diversity and richness. Paired *T*-test (LP vs. EAP) or independent *T*-test (HPC vs. LPC) was used after accessing normality with Shapiro–Wilk *W*-test. Data are presented as mean ± SD (*n* = 6), ^∗^*P* < 0.05, ^∗∗^*P* < 0.01, ^∗∗∗^*P* < 0.001.

In total, 24 fecal samples were used to perform 16S rRNA high-throughput sequencing using the Illumina HiSeq 2500 platform. On average, 62136 tags were verified, and 1183 OTUs per sample with 97% sequence similarity were obtained. Diversity differences between groups were accessed. Based on Chao1 and ACE, LPC sows contained significantly more observed species and higher microbial richness than HPC sows at LP stage (ACE, *P* < 0.001; Chao1, *P* < 0.01). At EAP stage, the microbial diversity (represented by Shannon and Simpson) of HPC sows was significantly higher than LPC sows (Shannon, *P* < 0.01; Simpson, *P* < 0.05) (Figure 1).

### Composition Changes of Gut Microbiota

**FIGURE 2:**
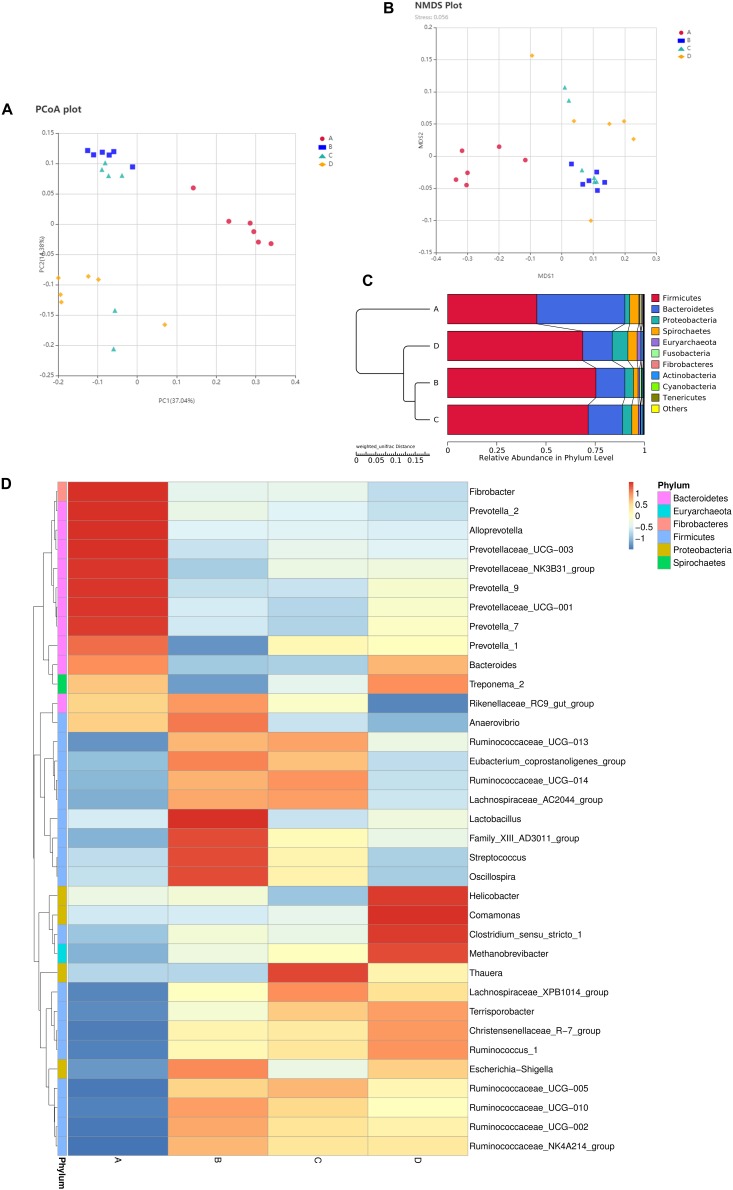
Composition differences of gut microbiota in sows. **(A,B)** Principal coordinate analysis (PCoA) and non-metric multidimensional scaling (NMDS) analyses. **(C)** Unweighted pair-group method with arithmetic mean (UPGMA) analysis. **(D)** Heatmap showing significantly different genera among groups. *n* = 6. A,C: sows with high productive capacity (HPC) at late pregnancy (LP) and early stage after parturition (EAP) separately; B,D: sows with low productive capacity (LPC) at late pregnancy (LP) and early stage after parturition (EAP) separately.

To evaluate the fecal microbial differences caused by reproductive capacities, we compared β-diversity and composition of the four groups. Results of the PCoA analysis based on Bray–Curtis dissimilarity and non-metric multidimensional scaling (NMDS) showed distinct separation patterns of group A and group B as well as group C and group D (Figures 2A,B), which suggested distinct microbial differences between LPC and HPC sows. Unweighted pair-group method with arithmetic mean (UPGMA) results disclosed that *Firmicutes*, *Bacteroidetes*, *Proteobacteria*, and *Spirochaetes* were the predominant floras (Figure 2C). The relative abundance of *Firmicutes* was accounted for at least 50% followed by *Bacteroidetes*, *Proteobacteria* and *Spirochactes*. Heatmap tree was used to show genera differences among groups and their phylogenic relationships (Figure 2D). For HPC sows, gut microbiota was mainly enriched in genera belonging to *Prevotellaceae* at LP stage and genera belonging to *Ruminococcaceae* at EAP stage. For LPC sows, genera belonging to *Firmicutes* (*Lactobacillus, Family_XIII_AD3011_group, Streptococcus, Oscillospira*) were the predominant microflora at LP stage, while these four genera were decreased at EAP stage.

**FIGURE 3:**
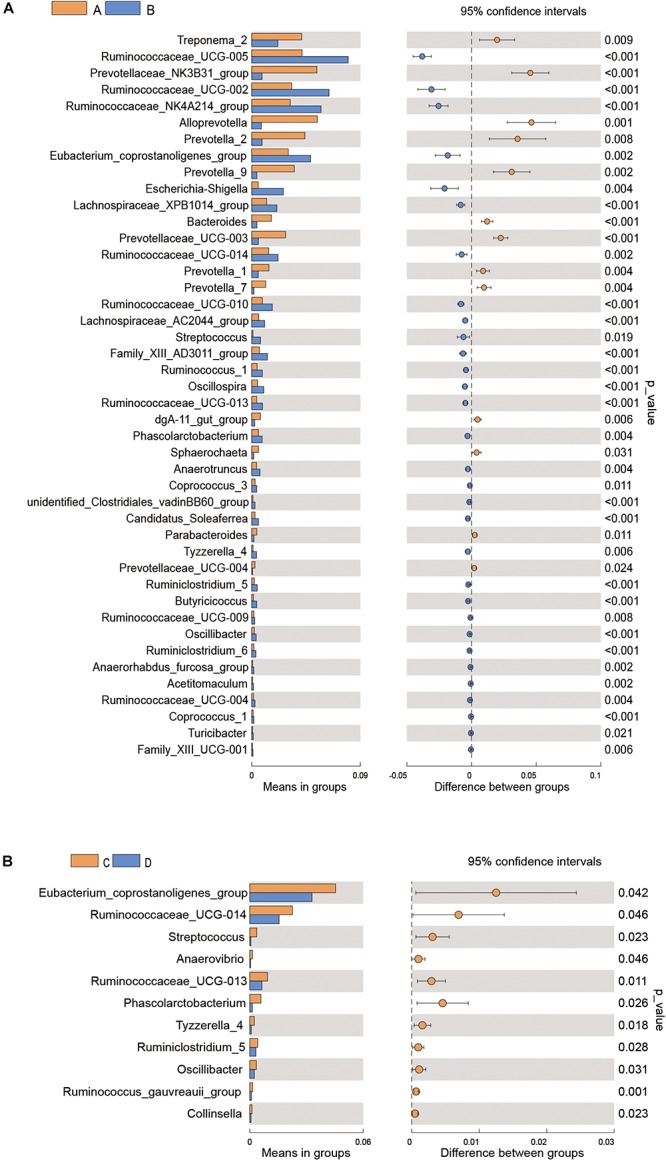
*T*-test bar plot of significantly different gut microbial species in sows at genus level **(A,B)**. *n* = 6. A, C: sows with high productive capacity (HPC) at late pregnancy (LP) and early stage after parturition (EAP) separately; B, D: sows with low productive capacity (LPC) at late pregnancy (LP) and early stage after parturition (EAP) separately.

**FIGURE 4:**
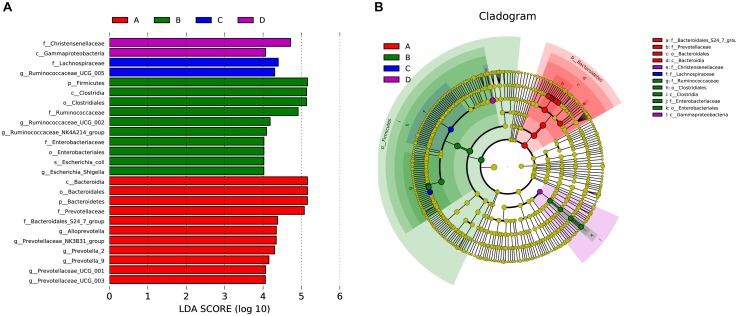
LEfSe analysis of gut microbial composition in sows with different productive capacities during perinatal period; **(A)** Histogram of the LDA scores, showing the biomarker taxa (LDA score > 4). **(B)** Cladogram obtained from LEfSe analysis, indicating the phylogenetic distribution of microbiota. *n* = 6. A, C: sows with high productive capacity (HPC) at late pregnancy (LP) and early stage after parturition (EAP) separately; B, D: sows with low productive capacity (LPC) at late pregnancy (LP) and early stage after parturition (EAP) separately.

Differences in gut microflora between groups at the genus level were explored using *T*-test bar plots. At LP stage, the relative abundance of *Prevotellaceae_NK3B31_group, Alloprevotella* and *Prevotella-2* in HPC sows was significantly higher while the relative abundance of *Ruminococcaceae_UCG-005, Ruminococcaceae_UCG-002* and *Ruminococcaceae_NK4A214_group* was significantly lower than LPC sows (Figure 3A). Further, the relative abundance of *Eubacterium_coprostanoligenes_group, Ruminococcaceae_UCG-014*, and *Phascolarctobacterium* in HPC sows was significantly higher than LPC sows at EAP stage (Figure 3B). In addition, gut microbial compositions were analyzed using the linear discriminant analysis (LDA) effect size (LEfSe) method (Figure 4), the results of which coincided with the *T*-test results, suggesting significant gut microbial differences between sows with different reproductive capacities during different stages of perinatal period.

### Metabolic Functional Changes of Gut Microbiota

**FIGURE 5:**
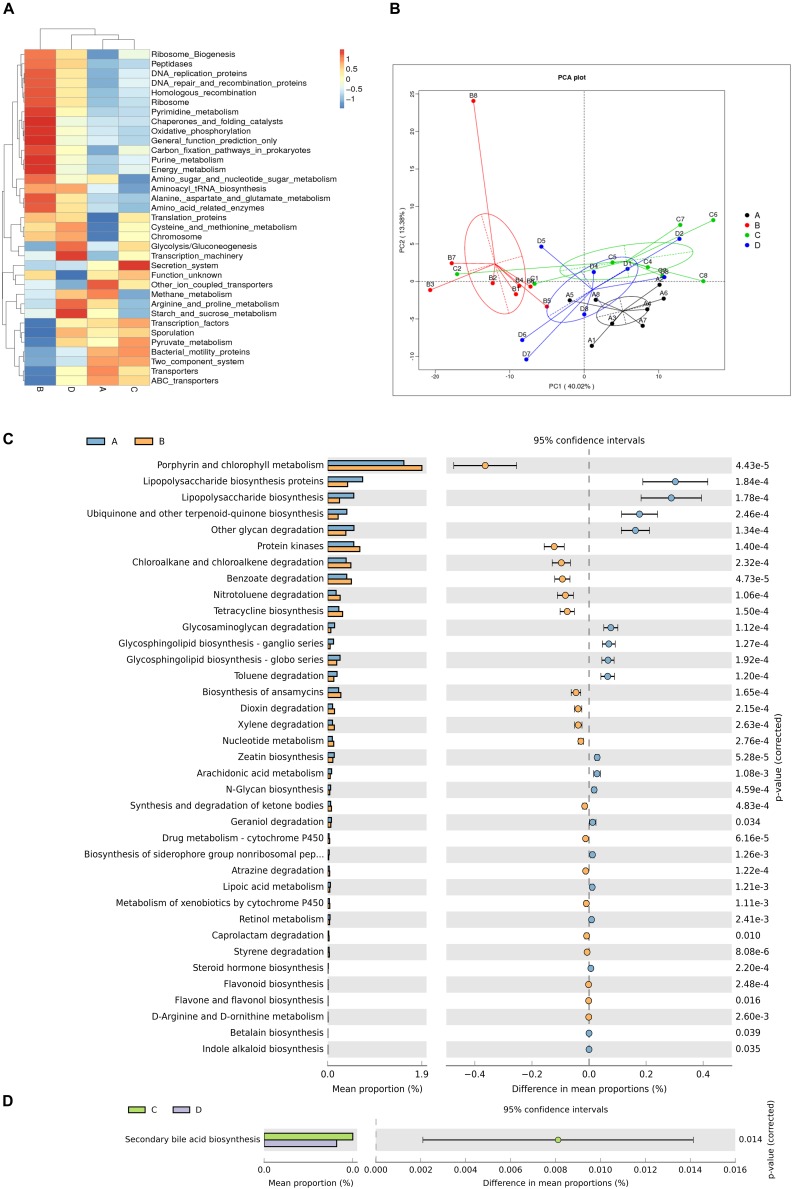
Differences in metabolic functions of gut microbiota. **(A)** Heatmap showing significantly different functional pathways. **(B)** Principal components analysis (PCA) plot of functional profiles among groups. **(C,D)** *T*-test bar plot of significantly differed metabolic pathways. *n* = 6. A, C: sows with high productive capacity (HPC) at late pregnancy (LP) and early stage after parturition (EAP) separately; B, D: sows with low productive capacity (LPC) at late pregnancy (LP) and early stage after parturition (EAP) separately.

Based on the significant differences in bacteria composition, we analyzed metabolic functional changes. PICRUSt was applied to produce metagenome based on 16S rRNA sequencing results at KEGG taxonomy level 3. PCA analysis based on KEGG annotation demonstrated clear clustering between group A and group B as well as group C and group D (Figure 5B). In addition, the heatmap showed the distributions of significantly different functional pathways among groups (Figure 5A), of which differential pathways related to microbial metabolism were selected.

Microbial gene functions related to metabolic pathways such as Lipopolysaccharide biosynthesis were significantly higher while pathways such as Porphyrin and chlorophyll metabolism and Protein kinases were significantly lower in HPC sows compared to LPC sows at LP stage (Figure 5C). Microbial gene functions related to secondary bile acid biosynthesis were also higher in HPC sows than LPC sows at EAP stage (Figure 5D).

### Metabolic Phenotypic Changes in Gut Microbiota

**FIGURE 6:**
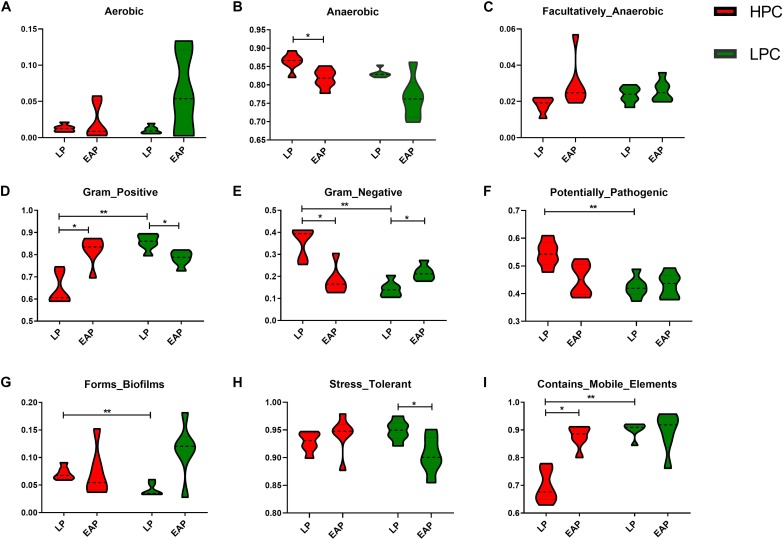
Metabolic phenotypes of gut microbiota from high productive capacity (HPC) and low productive capacity (LPC) sows at late pregnancy (LP) and early stage after parturition (EAP) stage. **(A–I)** Different indices included in microbial phenotypes. Wilcoxon signed-rank test (LP vs. EAP) or Mann–Whitney *U*-test (HPC vs. LPC) was applied. ^∗^*P* < 0.05, ^∗∗^*P* < 0.01 (*n* = 6).

To explore differences in bacterial metabolic phenotypes between HPC and LPC sows, BugBase was used and the results are shown in Figure 6. For gram stain, the relative abundance of gram-negative and gram-positive bacteria was significantly higher and lower (*P* < 0.01), respectively, in HPC sows than LPC sows at LP stage. Moreover, biofilm formation and potentially pathogenic capacity of HPC sow gut microflora were significantly higher (*P* < 0.01) than LPC sows at LP stage.

### Serum Biochemical Indices and Correlation With Gut Microbial Abundance in Sows

**FIGURE 7:**
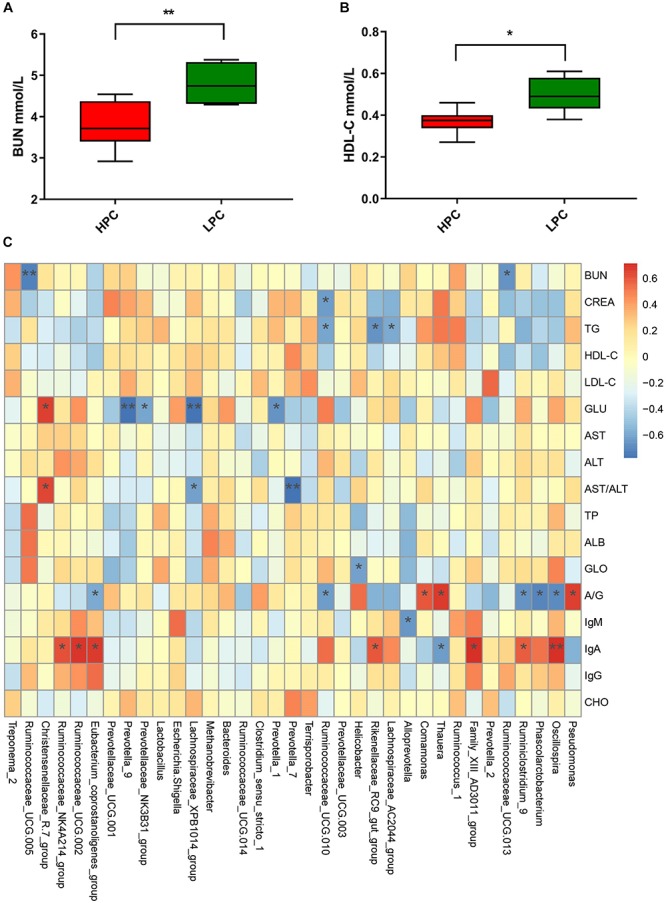
Serum biochemical indices differences in sows and correlations with gut microbial abundance during early stage after parturition. **(A)** Blood urea nitrogen (BUN, *n* = 6). **(B)** Serum high-density lipoprotein cholesterol (HDL-C, *n* = 6). **(C)** Heatmap of correlations between gut microbial abundance and serum biochemical indices at genus level. Paired *T*-test (LP vs. EAP) or independent *T*-test (HPC vs. LPC) was used after accessing normality with Shapiro–Wilk *W*-test to analyze serum biochemical indices differences. The correlations between microbial abundance at the genus level and serum biochemical indices were evaluated by Spearman’s correlation analysis. ^∗^*P* < 0.05, ^∗∗^*P* < 0.01. HPC, high productive capacity; LPC, low productive capacity; BUN, blood urea nitrogen; CREA, creatinine; TG, triglyceride; HDL-C, high-density lipoprotein cholesterol; LDL-C, low-density lipoprotein cholesterol; GLU, glucose; AST, aspartate aminotransferase; ALT, alanine aminotransferase; TP, total protein; ALB, albumin; GLO, globulin; A/G, ration of albumin/globulin; CHO, cholesterol.

Gut microbiota participates in the regulation of host’s immunity. Thus, we analyzed serum biochemical indices and their correlations with fecal microbial abundance. R software was used to perform Spearman correlation analysis of gut microbiota at the genus level at EAP stage (Figure 7C). BUN and HDL-C levels of HPC sows were significantly lower than LPC sows after parturition (Figures 7A,B). Further, the BUN level showed negative correlations with some genera belonging to *Ruminococcaceae* (*Ruminococcaceae_UCG-013, r* = *−*0.66, *P* < 0.05*; Ruminococcaceae_UCG-005, r* = *−*0.72, *P* < 0.01).

## Discussion

Composition, activity, and coevolution of gut microbiota with hosts are of great importance to animal health (Elson and Alexander, 2015; Woo and Alenghat, 2017). Remarkable changes occur in sow gut microbiota during pregnancy and lactation (Cheng et al., 2018; Liu et al., 2019). However, little is known about whether sows with different reproductive capacities have the same gut microbial performance during perinatal period. Therefore, we first investigated the large gut microbial variances between HPC sows (litter size ≥ 15) and LPC sows (litter size ≤ 7) as well as the correlation with serum biochemical indices during late pregnancy and early stage after parturition.

*Bacteroidetes, Firmicutes, Proteobacteria, and Actinobacteria* were the most abundant phyla in most mammals (Ley et al., 2008), consistent with our study that *Firmicutes* and *Bacteroidetes* were the most abundant phyla in all groups (Figure 2C). In this study, the microbial diversity of HPC sows was significantly higher than LPC sows at EAP stage. Previous study reported that higher diversity of gut microbiota represented greater plasticity in response to perturbations (Bäckhed et al., 2005; Kitano and Oda, 2006) and has been used as a health indicator (Shanahan, 2010). Thus, the significant gut microbial diversity of HPC sows may contribute to effective management of various disturbances during lactation. However, the gut microbiota richness of HPC sows was significantly lower than that in LPC sows at LP stage. Lower microflora richness has been linked to insulin resistance, dyslipidemia, and inflammation (Le Chatelier et al., 2013) and is also a significant marker of gut health (Lozupone et al., 2012). The results indicated that HPC sows might experience greater inflammation at LP stage. However, a pro-inflammatory environment is favorable for the contraction of the uterus, expulsion of the baby, and rejection of the placenta during the late pregnancy (Mor and Cardenas, 2010). Thus, the α-diversity differences might lead to greater plasticity to perturbations and a favorable environment for parturition in HPC sows.

We found that *Bacteroidetes* and *Firmicutes* were the most abundant phyla in all groups. The relative abundance of *Firmicutes* and *Bacteroidetes* is associated with energy metabolism of their hosts (Ley et al., 2005, 2006; Komaroff, 2017). These two bacteria might enhance intestinal function to meet energy needs for parturition. Compared to LPC sows, HPC sows had greater relative abundance of *Bacteroidetes* at LP stage and *Firmicutes* and *Bacteroidetes* at EAP stage. The differences in microflora might potentially contribute to distinct reproductive capacity due to their functions in energy metabolism. At the genus level, the relative abundance of *Alloprevotella*, *Prevotella-2*, and *Prevotellaceae_NK3B31_group* bacteria in HPC sows was higher while the relative abundance of *Ruminococcaceae_UCG-005, Ruminococcaceae_UCG-002* and *Ruminococcaceae_NK4A214_group* was significantly lower than LPC sows at LP stage. *Prevotellaceae* is correlated with amino acids, energy and vitamins metabolism (Zhang et al., 2018). *Ruminococcaceae* is negatively associated with Lipopolysaccharide (LPS) biosynthesis and lower *Ruminococcaceae* might contribute to inflammatory environment. However, inflammation during LP stage might contribute to the initiation of pregnancy (Mor and Cardenas, 2010; Kang et al., 2017). Differences of *Prevotellaceae* and *Ruminococcaceae* might provide better condition for farrowing and promote fetal growth at LP stage. The relative abundance of *Eubacterium_coprostanoligenes_group* and *Ruminococcaceae_UCG-014* in HPC sows was significantly higher than LPC sows at EAP stage (Figure 3B). *Eubacterium* can produce SCFAs from amino acids and SCFAs possess anti-inflammatory effects (Kanauchi et al., 1999; Tedelind et al., 2007). Scarpa et al. (2011) reported that *Eubacteriaceae* was negatively related to polymorphonuclear cell and monocyte infiltration. Further, butyrate-producing *Ruminococcaceae* could reduce LPS biosynthesis (Kang et al., 2017), suggesting that HPC sows might suffer less inflammation at EAP stage, which might contribute to postpartum recovery.

PICRUSt was used to analyze metabolic functional changes. Microbial gene functions related to metabolic pathways such as LPS biosynthesis were significantly higher in HPC sows compared to LPC sows at LP stage. LPS is a component of the cell walls of gram-negative bacteria, which could lead to severe inflammation by upregulating the expression of interleukin-1 and tumor necrosis factor in the lung (Ulich et al., 1991). PICRUSt results indicated that HPC sows suffered greater inflammation than LPC sows. Conversely, LPS plays a role in the adhesion of microflora to gut mucosa (Nevola et al., 1985), and it is important for the activation of immune responses (Takeda and Akira, 2005). Moreover, LPS contributes to a pro-inflammatory environment, which promotes the initiation of parturition (Norman et al., 2007). These results indicated that gut microbiota in HPC sows might lead to a more favorable physiological status for parturition than LPC sows at LP stage. Microbial gene functions related to metabolic pathways such as secondary bile acid biosynthesis in HPC sows were significantly higher compared to LPC sows at EAP stage. Secondary bile acids generated by gut microbial enzymes are important signaling molecules and metabolic regulators to host’s pathways (Valdes et al., 2018). Low concentrations of secondary acids exhibit anti-inflammatory effect by reducing pro-inflammatory cytokines while high concentrations can cause DNA damage, oxidative stress and apoptosis (Ajouz et al., 2014; Ward et al., 2017a). The underlying cause of the secondary bile acids variances between LPC and HPC sows remains to be further elucidated.

There were significant differences in gut microbial metabolic phenotypes between sows with different reproductivity during different periods. The relative abundance of gram-positive and anaerobic bacteria in HPC sows was significantly higher than LPC sows at LP stage, which might be a marker for sows with different reproductivity at LP stage. Further, stress-tolerant bacteria were more abundant in HPC sows than LPC sows at EAP stage, indicating that HPC sows could better handle various stresses after parturition. Further, it has been reported that bacterial genes coding cell surface proteins (including lipopolysaccharide biosynthesis proteins) play a role in biofilm formation (Theunissen et al., 2010). The relative abundance of biofilm forming bacteria in HPC sows was significantly higher than LPC sows at LP stage (Figure 6), which was consistent with more metabolic pathways related to LPS biosynthesis in HPC sows (Figure 5C). Correspondingly, the biofilm formation of bacteria is associated with drug resistance (Stewart and Costerton, 2001). However, the mechanism of biofilm formation differences remains to be further studied. Further, there were higher abundances of potentially pathogenic bacteria in HPC sows than LPC sows, which might lead to a pro-inflammatory environment at LP stage.

Blood urea nitrogen is the final catabolism product of proteins, which could reflect the amino acids balance (Coma et al., 1995). HPC sows had lower BUN levels, indicating that amino acids in HPC sows were more balanced than LPC sows. Further, BUN level showed a negative correlation with *Ruminococcaceae*. *Ruminococcaceae* is related to amino acids metabolism (Zhang et al., 2018). Thus, the BUN differences might be caused by *Ruminococcaceae.* High-density lipoprotein cholesterol is negatively related to cardiovascular disease (Gordon et al., 1989), and it promotes prostaglandin I_2_ synthetase activity (Beitz and Förster, 1980). HDL-C level decreases after parturition, which may be affected by hormonal, body composition or life-style changes (Lewis et al., 1996). The mechanism underlying the difference in HDL-C levels between groups needs to be further explored.

## Conclusion

We found tremendous microbial diversity, composition, metabolic functions, phenotypes and serum indices differences between HPC and LPC sows during perinatal period, especially at the LP stage. Microbial richness was significantly lower at LP stage, while microbial diversity was significantly higher at EAP stage in HPC sows. Additionally, there were also significant differences in BUN and HDL-C levels after parturition. This study discloses great microbial differences between HPC and LPC sows during perinatal period, which might lead to an inflammatory environment at LP stage and an anti-inflammatory environment at EAP stage, and these differences might promote high productive capacity. However, further studies are needed to explain causes of the microbial differences and their relationships with productive capacity.

## Data Availability Statement

The datasets generated for this study can be found in the NCBI Sequence Read Archive (No. PRJNA565644).

## Ethics Statement

The animal study was reviewed and approved by the Animal Welfare Committee of the Institute of Subtropical Agriculture, Chinese Academy of Sciences.

## Author Contributions

JZ, XX, LZ, XK, BT, and YY designed the study. JZ and XX carried out the animal trials and sample analysis. YS, JZ, and XX wrote and revised the manuscript.

## Conflict of Interest

The authors declare that the research was conducted in the absence of any commercial or financial relationships that could be construed as a potential conflict of interest.

## Footnotes

1 http://greengenes.lbl.gov/Download/

2 http://sourceforge.net/projects/rdp-classifier/

3 https://www.genome.jp/kegg/ko.html

4 https://bugbase.cs.umn.edu/
