# Supplementary material for: Differences in Gut Microbial and Serum Biochemical Indices Between Sows With Different Productive Capacities During Perinatal Period
Source: Front Microbiol. 2020 Jan 17;10:3047. doi: 10.3389/fmicb.2019.03047 (PMC6978668; doi:10.3389/fmicb.2019.03047)
Supplement: Supplementary file 1 [file Table_1.DOCX]

***Supplementary Material***

**Table S1. Litter sizes of each low productive capacity (LPC) and high productive capacity (HPC) sows**

|  |  |
| --- | --- |
| **Group** | **Litter size** |
| LPC | 5 |
|  | 5 |
|  | 5 |
|  | 5 |
|  | 4 |
|  | 5 |
|  |  |
| HPC | 17 |
|  | 15 |
|  | 16 |
|  | 16 |
|  | 15 |
|  | 16 |
